# Green Vertical-Cavity Surface-Emitting Lasers Based on InGaN Quantum Dots and Short Cavity

**DOI:** 10.1007/s40820-023-01189-0

**Published:** 2023-10-09

**Authors:** Tao Yang, Yan-Hui Chen, Ya-Chao Wang, Wei Ou, Lei-Ying Ying, Yang Mei, Ai-Qin Tian, Jian-Ping Liu, Hao-Chung Guo, Bao-Ping Zhang

**Affiliations:** 1 Laboratory of Micro/Nano-Optoelectronics, School of Electronic Science and Engineering, Xiamen University, Xiamen, 361005 Fujian People’s Republic of China; 2 Suzhou Institute of Nano-Tech and Nano-Bionics, Chinese Academy of Sciences, Suzhou, 215123 Jiangsu People’s Republic of China; 3 Department of Photonics, National Yang Ming Chiao Tung University, Hsinchu, 30010 Taiwan People’s Republic of China; 4 Semiconductor Research Center, Honhai Research Institute, New Taipei, 220236 Taiwan People’s Republic of China

**Keywords:** Green vertical cavity surface emitting laser, GaN, Low threshold, InGaN quantum dots

## Abstract

Room temperature low threshold lasing of green GaN-based vertical cavity surface emitting laser (VCSEL) was demonstrated under continuous wave (CW) operation. By using self-formed InGaN quantum dots (QDs) as the active region, the VCSEL emitting at 524.0 nm has a threshold current density of 51.97 A cm^−2^, the lowest ever reported. The QD epitaxial wafer featured with a high IQE of 69.94% and the δ-function-like density of states plays an important role in achieving low threshold current. Besides, a short cavity of the device (~ 4.0 λ) is vital to enhance the spontaneous emission coupling factor to 0.094, increase the gain coefficient factor, and decrease the optical loss. To improve heat dissipation, AlN layer was used as the current confinement layer and electroplated copper plate was used to replace metal bonding. The results provide important guidance to achieving high performance GaN-based VCSELs.

**Supplementary Information:**

The online version contains supplementary material available at 10.1007/s40820-023-01189-0.

## Highlights

- Continuous-wave green vertical-cavity surface-emitting lasers based on self-formed quantum dots were realized with the lowest threshold current density of 51.97 A cm^−2^.
- A short cavity (~4.0 λ, where λ is the wavelength in the media) was adopted to enhance the interaction between spontaneous emission and lasing mode, with a big coupling factor up to 0.094.
- AlN current confinement layer and the electroplated supporting copper plate were utilized to improve heat dissipation, with a low thermal resistance of 842 K W^−1^.

**Supplementary Information:**

The online version contains supplementary material available at 10.1007/s40820-023-01189-0.

## Graphical abstract

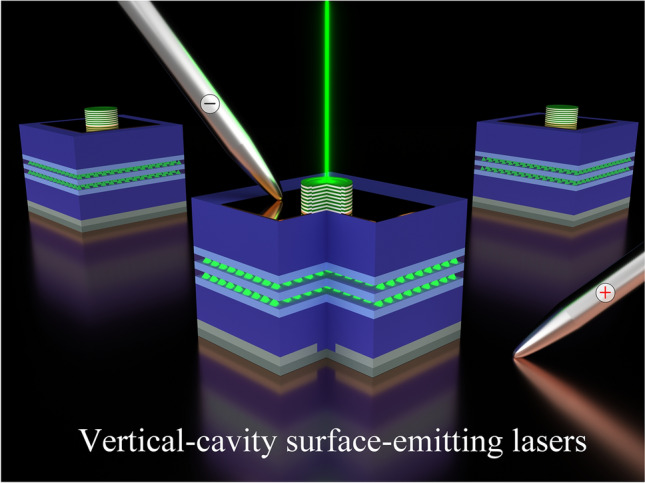

## Introduction

Vertical cavity surface emitting lasers (VCSELs) was proposed by K. Iga in 1977 [1] and, compared with edge emitting semiconductor lasers (EELs), have the characteristics of circular beam, small size, low threshold, and easy integration etc. [2–4]. In the infrared spectral range, GaAs-based VCSEL was commercialized in 1997 in mainly data communication systems [5]. In recent years, it has also become key devices in application fields [5] such as 3D perception and autonomous driving, and has received high attention from both the scientific research [6, 7] and the industry [8, 9] as well. In the visible spectral range, GaN-based VCSELs are also in the spotlight of GaN optoelectronic research because of their potential applications in visible light communication, biochemical sensing, high-resolution laser printing/scanning, display, and data storage [10–12]. In recent years, GaN-based VCSELs have been successfully demonstrated by both academic [13–17] and industrial [18–21] research groups. However, the reported GaN-based VCSELs are mainly in the violet and blue spectra region, and there are only a few reports about green VCSELs [22–27]. Two-dimensional (2D) InGaN quantum wells (QWs) are typically utilized as the active region for GaN-based VCSELs. For devices emitting in the green, InGaN QWs with higher indium (In) content are needed. However, increasing In content will lead to stronger quantum confinement Stark effect (QCSE) and a higher density of defects due to the large lattice mismatch between GaN and InGaN [28]. In addition, the large effective mass of carriers in the GaN-based material system results in a higher transparent carrier density, which is another limitation to achieving low threshold green GaN-based VCSELs [29]. At present, the electrically injected green VCSELs based on c-plane InGaN QWs are only realized by Nichia in 2011 with double dielectric distributed Bragg reflector (DBR) structure [22], and in 2021 with hybrid DBR structure [27], respectively. For their green VCSEL with hybrid DBR structure, lattice-matched AlInN/GaN DBR were grown on the c-plane GaN substrate, and lasing at 514.9 nm with a threshold current density of 14.3 kA cm^−2^ was realized [27]. On the other hand, growing InGaN QWs on semipolar or nonpolar GaN can decrease the QCSE and improve the emission efficiency in green spectral region [30, 31]. In 2020, Sony utilized (20-21) semipolar GaN substrate to grow InGaN/GaN MQWs and achieved lasing of green VCSEL at 515 nm, but the threshold current density is still relatively large of 14.4 kA cm^−2^ [26].

Using Quantum dots (QDs) as the active region is an effective approach to overcome problems associated with QWs [23, 24]. In the growth of QDs by Stranski–Krastanow growth mode, the driving force of QD formation is the strain existing in the film. The QD growth is accompanied by strain relaxation. Then, the piezoelectric polarization field in the QD and the QCSE are almost eliminated [32]. QDs are zero-dimensional materials in which electrons and holes are well confined in a small space, thus forming the δ-function-like density of states, which is important for achieving low threshold current density [33–35]. Meanwhile, the strong localization effect of QDs can effectively prevent carriers from being captured by nonradiative recombination centers and improve the emission efficiency of the active region [36].

In our previous work, using QD-based active region, we successfully achieved low threshold green VCSELs emitting from 491.8 to 565.7 nm, and the threshold current is in the order of sub-milliampere [24]. For those VCSELs, a SiO_2_ insulator layer was used as current confinement and metal bonding was adopted to transfer the VCSEL structure on a copper (Cu) plate. Unfortunately, the SiO_2_ material with low thermal conductivity (1.5 W mK^−1^) [37] can cause poor heat dissipation because it is located in the main pathway of thermal conduction [38]. In addition, the VCSEL with a metal bonding substrate also faces thermal dissipation problems because cracks and air voids or gaps are easily formed at the bonding interface [39–41]. Meanwhile, the long cavity (11 ~ 15 λ, where λ is the wavelength in the media) of device can also induces large scattering and absorption losses.

In this study, InGaN QD-based green VCSELs were fabricated with optimized fabrication processes. The copper supporting plate of the device was directly formed by electroplating process instead of metal bonding, and a buried AlN current confinement layer was utilized instead of SiO_2_ to improve the heat dissipation while maintaining current confinement properties. Simultaneously, due to the smaller refractive index of SiO_2_ compared with GaN, positive optical guiding was realized [42]. The positive index-guiding structure also helps to suppress the lateral optical leakage of the resonating modes. Moreover, a much shorter cavity (~ 4.0 λ) was used to further decrease the internal loss and enhance the spontaneous emission coupling factor. Finally, room temperature continuous-wave (CW) lasing with the lowest threshold current density of 51.97 A cm^−2^ was realized at 524 nm. These results provide a guideline for high performance green GaN-based VCSELs.

## Materials and Fabrication

The epitaxial wafer was grown on a c-plane (0001) sapphire substrate by MOCVD system. The InGaN QD layers were grown as active region by the Stranski–Krastanow growth mode. In the growth process, Triethylgallium (TEGa) and Trimethylindium (TMIn) were used as precursors for Ga and In sources, respectively, for growing InGaN layers. Ammonia gas (NH_3_) was used as precursor for N source. Hydrogen (H_2_) was used as the carrier gas for growing GaN template, while nitrogen (N_2_) for QDs. The InGaN QDs were deposited at 670 °C with a molar gas phase ratio, TMIn/(TMIn + TEGa) of about 1:2, and the V/III ratio was set to be 1.35 × 10^4^. After the deposition of QDs, a two-step growth was used to grow the GaN cap layers. First, a 2-nm-thick low-temperature grown GaN layer was deposited at the same growth temperature (670 °C) as QDs to protect them during subsequent temperature ramping process. Then, the temperature was ramped to 850 °C and an 8-nm-thick GaN barrier layer was grown. The active region consisted of two layers of InGaN/GaN QDs, the indium content of InGaN QDs is about 0.27. The Cross-section Z-contrast scanning transmission electron microscopy (STEM) shows a truncated pyramid shape of QD, which is a typical shape of QD grown by MOCVD [43]. The diameter ranges of QD from 20 to 60 nm with an average height of 2.5 nm, while the QD density is ~ 1.5 × 10^10^ cm^−2^ [43].

**Fig. 1:**
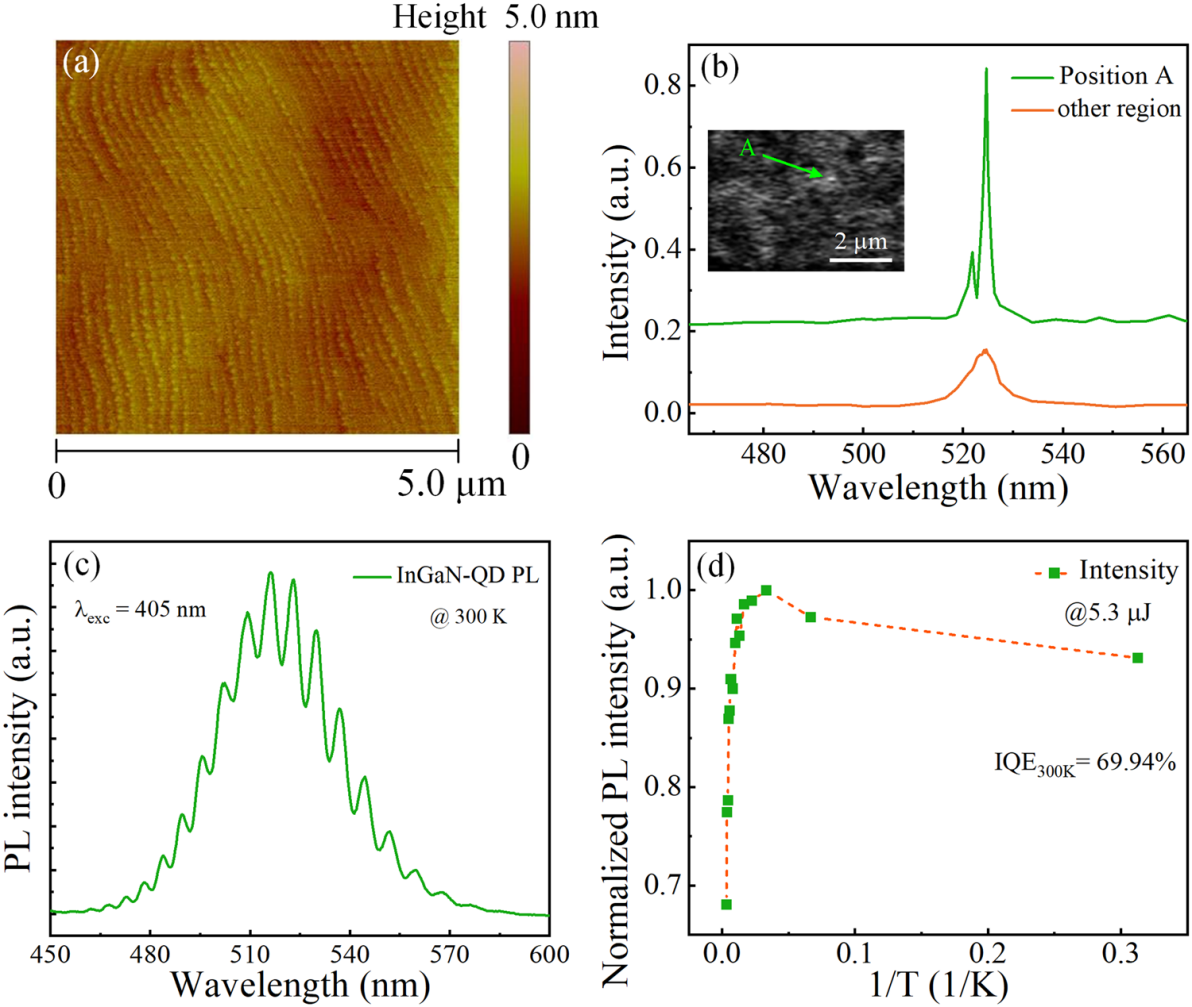
**a** 5 × 5 μm^2^ AFM image of the uncapped InGaN QD layer. **b** CL spectra from light spot A and other regions of the QD sample, inset shows the CL image at 4 K. **c** PL emission spectra of QD epitaxial wafer at 300 K. **d** Normalized integrated PL intensity as a function of 1/T for the InGaN QD emission

Figure 1a shows the 5 × 5 μm^2^ atomic force microscope (AFM) image of the uncapped InGaN QDs, which are perfectly aligned along the step edge. The light-emission properties of the QD wafer were also studied using spatially resolved spot-focus cathodoluminescence (CL) at low temperature (4 K), and CL spectra were measured through the 5-μm diameter apertures of a metal mask. For the CL image shown Fig. 1b, there is just only one QD, spot A. The diameter of bright spot A in the CL image is about 100 nm. The emission from spot A is narrower than the emission from “other region” where many dots are included. It is of course much narrower than the emission from an even large area (Fig. 1c). These sharp peaks show a δ-function-like emission line that are believed to come from the QD-like structure. The photoluminescence (PL) spectrum of the epitaxial wafer was measured under excitation of a diode laser (λ = 405 nm) at 300 K, which is depicted in Fig. 1c. The spontaneous emission of the QDs starts from 450 nm and ends at 600 nm, have a much wider FWHM (~ 43 nm) than QW. The broad spontaneous emission spectra are caused by the fluctuation of the indium content and the inhomogeneous size of the QDs. Figure 1d shows the normalized integrated emission intensities as a function of the reciprocal temperature for the QD sample. At lower temperature (3.2–30 K), the PL intensity was relatively large because the non-radiative recombination centers are frozen and inactivated, and consider that the internal quantum efficiency (IQE) is 100% at that temperature [44–47]. As the temperature rises, the PL intensity became smaller due to the non-radiative centers are thermally activated. The IQE was defined as the ratio of the integrated PL intensity at 300 and 15 K in this work, which has a relatively large IQE of 69.94%.

The QD epitaxial wafer featured with a large localization energy and a δ-function-like density of states, which bring about many advantages than QWs [48]. For example, the QDs have a higher differential gain than QWs, benefitting low-threshold lasing [49, 50]. In addition, the self-formed InGaN/GaN QDs are characterized by nearly zero internal electric field (then almost no QCSE) and consequently a higher electron–hole recombination probability [51, 52]. These effects have been demonstrated previously both theoretically [32, 53] and experimentally [49, 54]. Moreover, the increased confinement restrains carrier diffusion, making electrons and holes less susceptible to nonradiative recombination centers induced by defects [55]. Therefore, the emission and IQE are enhanced (~ 69.94%). These superior advantages of InGaN QD are very important to realize a low threshold current of VCSEL.

**Fig. 2:**
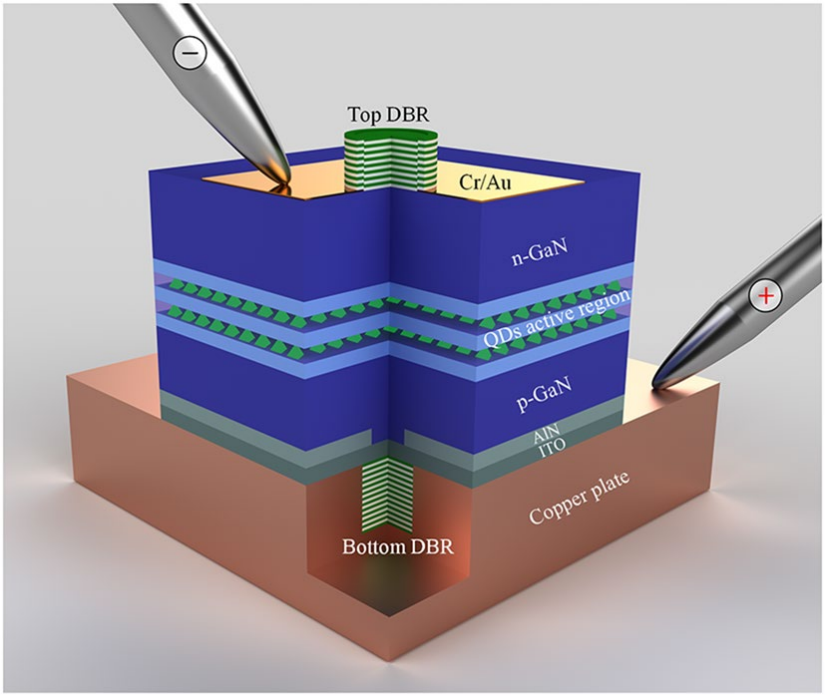
Schematic diagram of the GaN-based VCSEL with InGaN QD active region

**Fig. 3:**
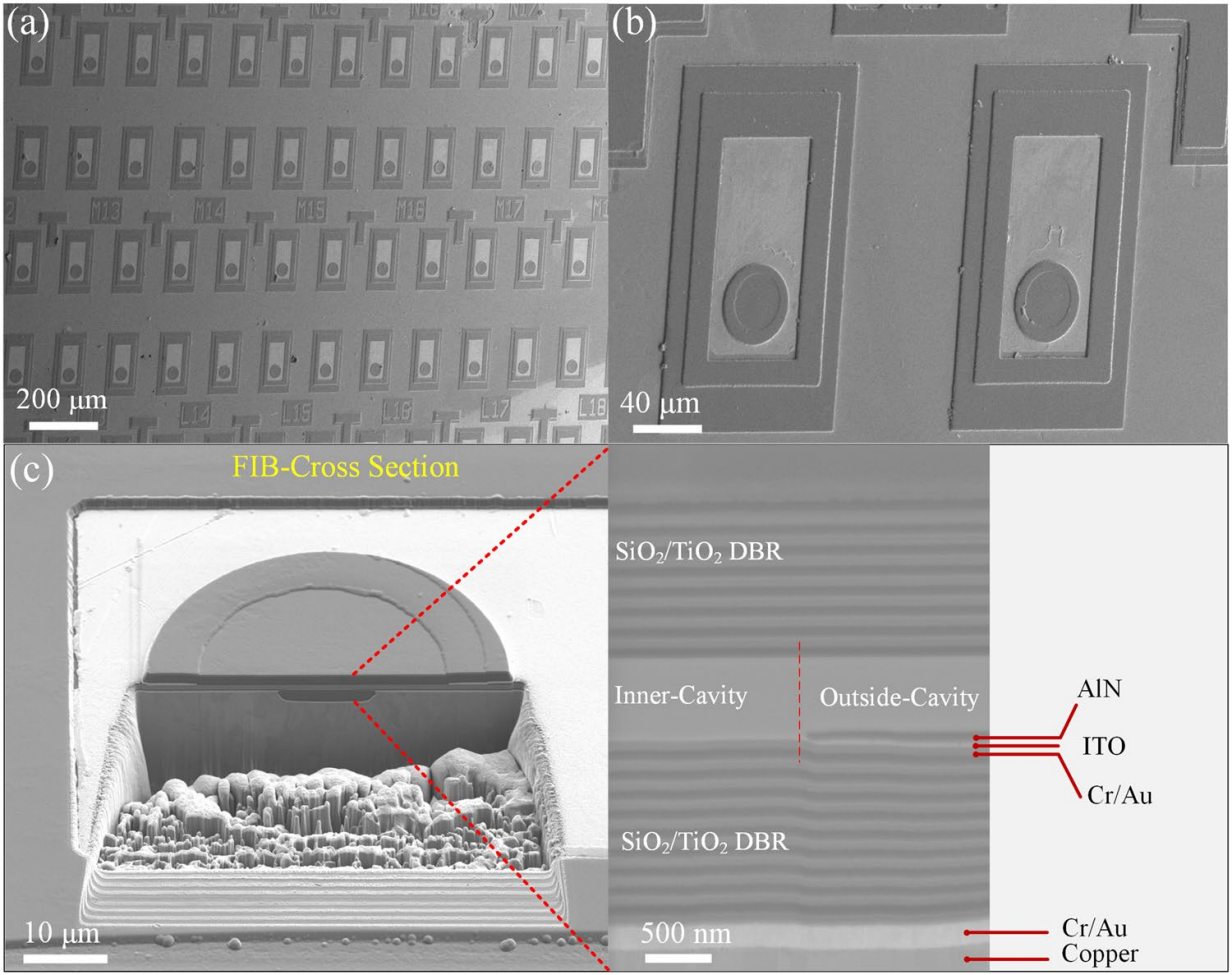
SEM image of the devices with different magnifications: **a** 50^×^, **b** 300^×^. **c** FIB cross-section SEM image of devices

The device structure of the VCSEL with a Cu supporting plate and dual dielectric DBRs is illustrated in Fig. 2. To fabricate the device, the p-GaN mesa with a diameter of 7 μm was firstly formed by inductively coupled plasma (ICP) etching. Secondly, an AlN insulating layer with 75 nm thickness was deposited by magnetron sputtering around the p-GaN mesa to form a current-confinement structure. To realize an intra-cavity contact structure, an Indium Tin Oxide (ITO) layer was evaporated on the upper surface of AlN confinement layer and p-GaN as a current spreading layer. Then, 12.5 pairs of TiO_2_/SiO_2_ bottom DBR were deposited and patterned. Subsequently, the Cr/Au p-electrode was deposited, and then a copper layer (~ 205 μm) was electroplated as the new supporting plate. After that, the sapphire substrate was detached by laser lift-off (LLO), and the n-side epilayers were thinned by ICP and chemical mechanical polishing (CMP). The device mesa was formed by ICP etching, after which the n-contact layer (Cr/Au) and 8 pairs of TiO_2_/SiO_2_ top dielectric DBR were finally deposited (For detailed fabrication of VCSEL, see Supplementary Information). The scanning electron microscope (SEM) of the devices was shown in Fig. 3a, b. The length and width of the device are 160 and 100 μm, respectively. Figure 3c is a focus ion beam (FIB) cross-section image, showing the sub-micron cavity between the top and bottom DBR. The current spreading ITO layer extending to the inner-cavity, and the AlN current confinement layer are clearly identified. The cooper plate exhibits good compactness without cracks compared with metal bonding, which ensures the heat dissipation performance of VCSEL.

## Results and Discussion

**Fig. 4:**
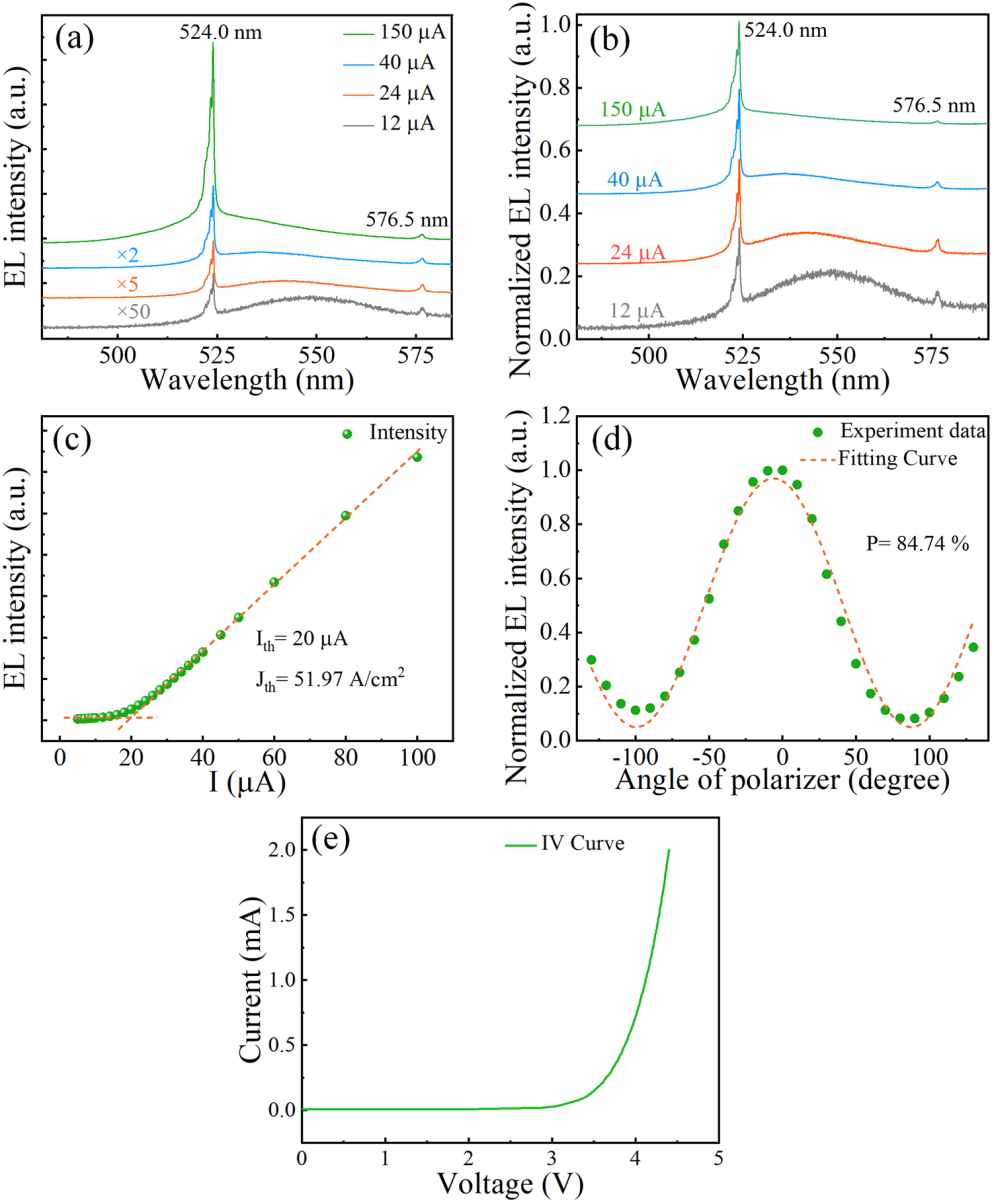
RT-CW lasing characteristics. **a** The amplified EL and **b** normalized EL spectra at different currents. **c** EL intensities as a function of currents. **d** Normalized EL intensities with varying angles of polarizer at 200 μA. **e I-V** characteristics of the VCSEL under CW operation at 300 K (The spectra with different currents in **a** and** b** are offset along the *y*-axis for clarity)

The optical characteristics of the device were measured under CW bias currents at RT. Figure 4a shows the electroluminescence (EL) spectra of the device under different injection currents. The spectra under smaller currents were numerically amplified for clarity. The wavelength of the main longitudinal mode is 524.0 nm and the intensity increases nonlinear with increasing current. Besides, another longitudinal-mode-related peak can be observed at 576.5 nm, the longitudinal mode spacing is 52.5 nm. The measurement results (See Supplementary Information) show that an average thickness of 879 nm of cavity (~ 4.0 λ). To better study the spectral variation at different currents, the normalized EL intensity was plotted in Fig. 4b. Below threshold, strong spontaneous emission can be observed from the spectrum. As the current increases, the evolution of spectra shows a transition from spontaneous emission to stimulated emission. Above threshold, the main mode at ~ 524.0 nm is gradually dominant with the suppression of spontaneous emission and the side modes. Figure 4c shows the EL intensity as a function of the current, which exhibits a threshold behavior at a low current of 20 μA (corresponding a current density ~ 51.97 A cm^−2^). The polarization characteristics of the VCSEL are shown in Fig. 4d, and a degree polarization of 84.74% under 200 μA was obtained, which is another evidence of lasing. Figure 4e shows the typical current–voltage (*I–V*) characteristics of the devices, and a turn-on voltage of 3.84 V is obtained.

Apart from QDs, the short cavity length is also critical in achieving low threshold lasing of InGaN QD VCSELs. The related physical mechanism can be attributed to the enhancement of the spontaneous emission coupling factor and the decrease of internal absorption loss. The spontaneous emission coupling factor can be significantly enhanced by 7.7 times when the cavity length was thinned from 18 to 6 λ in our previous work [56]. The spontaneous emission coupling factor (*β*) can be defined as the fraction of spontaneous emission coupled into a cavity mode with respect to the spontaneous emission into all modes [57], and depends on the Purcell factor of the cavity [56]:

1 $$\beta = \frac{{F_{p} }}{{1 + F_{p} }}$$

where Purcell factor *F*_*p*_ relates to the cavity length [56, 58]:

2 $$F_{p} \propto \frac{1}{{\left[ {\ln \left( {R_{1} R_{2} } \right)^{{{1 \mathord{\left/ {\vphantom {1 2}} \right. \kern-0pt} 2}}} + L_{c} \alpha_{i} } \right]}}$$

where *R*, *L*_*c*_, and *α*_*i*_ represent the reflectivity of top/bottom DBR (> 99.5%), cavity length, and absorption of VCSEL, respectively. The equations indicate that a shorter cavity length can benefits the *β*.

**Fig. 5:**
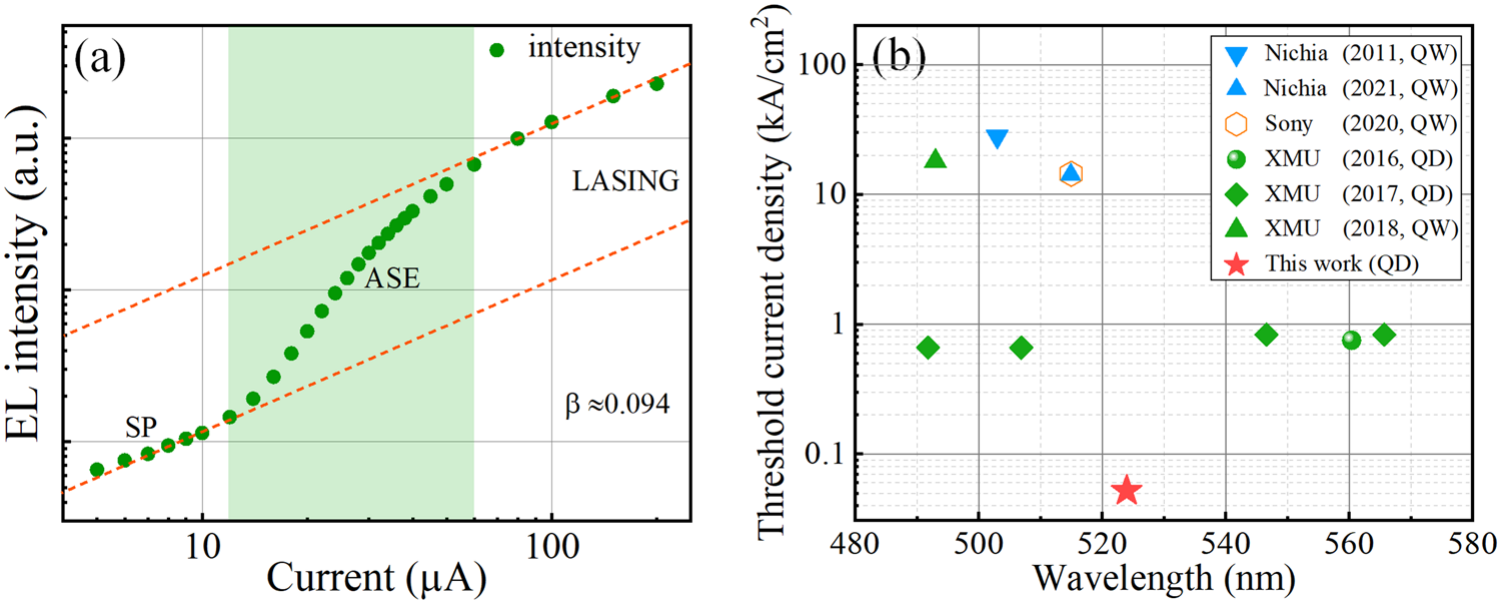
**a** EL intensity versus injection current in double logarithmic scale. **b** Threshold current densities and wavelengths of ever-reported electrically injected green GaN-based VCSELs so far

To extract the spontaneous emission coupling factor of the QD VCSEL here, the EL intensity versus the injection current was plotted in a double-logarithmic scale, as shown in Fig. 5a. The typical ‘S’ shape of the I-L curve includes the spontaneous emission (SE) region, the amplified SE (ASE) region, and the lasing region, which denote a standard lasing evolution process. The *β* was calculated to be 0.094, which is a large value for electrically injected VCSELs. The *β* of typical electrically injected VCSELs and EELs are normally about 10^–3^ and 10^–5^ [59–62], respectively. With the increasing value of *β*, the laser will be thresholdless when *β* is equal to 1 [57]. The value of 0.094 (94 × 10^–3^) means that 94 photons out of 10^3^ spontaneously emitted photons are coupled to a lasing mode and serve as a ‘seed’ for oscillation [63]. That is to say, a large *β* indicates that more spontaneous photons can be incorporated into the lasing mode, thus reducing the threshold current [64].

The short cavity in this study is also helpful in reducing internal optical loss, and the positive optical guiding effect is beneficial to reduce diffraction loss. Meanwhile, a short cavity can also enhance the gain coefficient factor (κ). The factor (κ) can be expressed as follow [56]:

3 $$\left\{ \begin{gathered} \kappa \approx \frac{{2\Delta \upsilon_{c} }}{\pi \gamma } \hfill \\ \Delta \upsilon_{c} \approx \frac{c}{{2nL_{c} }} \hfill \\ \end{gathered} \right.$$

where γ is the FWHM of the spontaneous emission without cavity, *n* is the refractive index of the resonant cavity, and Δυ_*c*_ is the frequency spacing between longitudinal modes. Consequently, the gain coefficient factor (κ) can be derived as:

4 $$\kappa \approx \frac{c}{{n\pi \gamma L_{c} }}$$

From the equation, the decreased cavity length can enhance the gain coefficient factor. Compared with our previous VCSELs (with a cavity length of about 2–3 μm) [24], the shorter cavity length (879 nm) in this work can increase the gain coefficient factor several times. Owing to a much shorter cavity, the decreased internal absorption loss, as well as the enhancement of spontaneous emission coupling factor and gain coefficient factor are achieved. All these are essential for low-threshold lasing.

The threshold current densities and lasing wavelengths of green VCSELs from different research groups are summarized in Fig. 5b. The threshold current density in this work is significantly lower than previously reported values. Compared with devices using InGaN QWs as the active region, the threshold current is reduced for more than two orders after using QDs. The VCSEL with a 7 μm diameter current aperture has a corresponding threshold current density of 51.97 A cm^−2^, which is a low value among the ever-reported GaN-based VCSELs [22–27], indicating the high potential of QDs.

Heat dissipation also plays an important role in determining properties of GaN-based VCSELs. High junction temperature can easily deteriorate material gain and device performance, eventually affecting the threshold current, emission spectrum, etc. [65, 66]. To improve thermal dissipation, an AlN current confinement layer with a higher thermal conductivity of (~ 200 W mK^−1^) [38] is used instead of SiO_2_ (1.5 W mK^−1^). In addition, the electroplated copper plate in this work can further promote heat conduction from the AlN layer to the heat sink. Unlike the metal bonding in our previous work [23, 24], the electroplated copper is uniform and dense, which can avoid the formation of cracks, air holes or gaps occurring easily in a bonding process [30, 67].

**Fig. 6:**
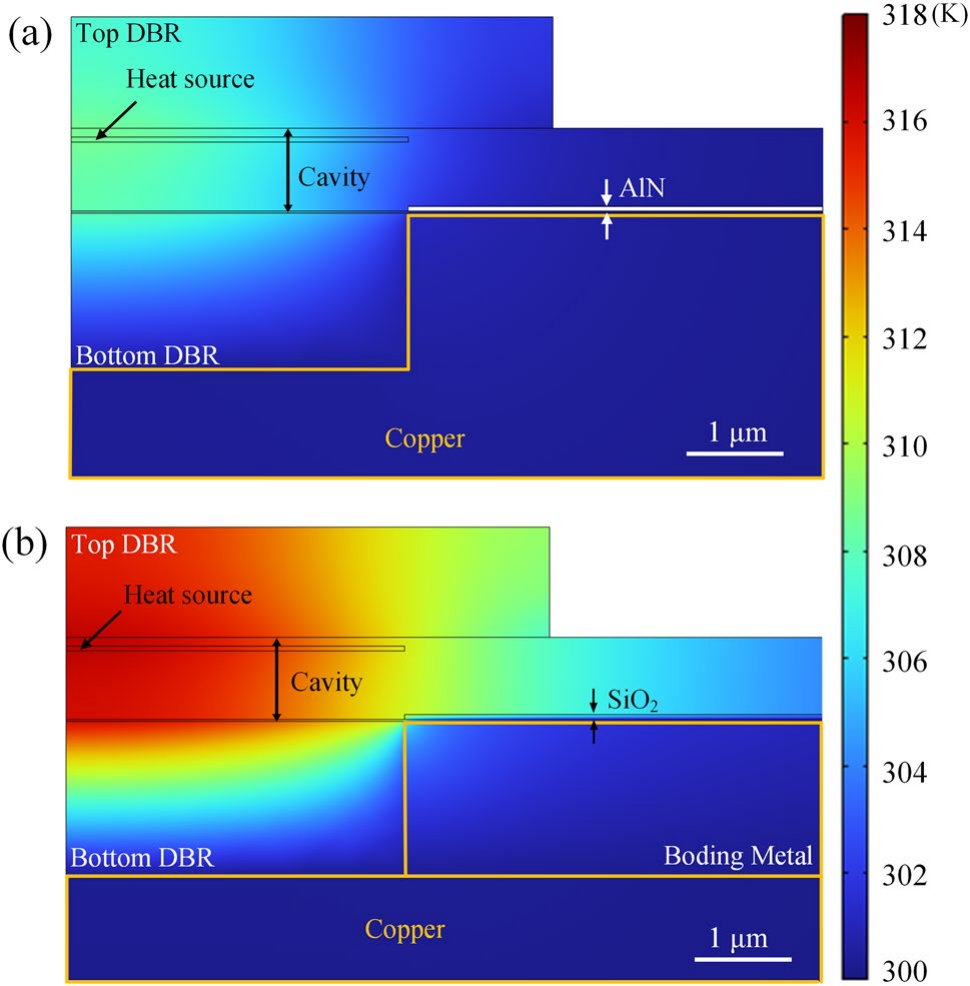
Temperature distribution of GaN-based VCSELs of **a** Structure A (using electroplated copper plate and AlN insulating layer) and **b** Structure B (using Cu-Sn bonding and SiO_2_ insulating layer)

To study the improvement of thermal dissipation, a steady-state quasi-3D heat dissipation model was used to calculate the temperature distribution of the VCSEL. Figure 6a shows the thermal profile of the device studied here with using the electroplated copper plate and AlN current confinement layer (defined as Structure A). For comparison, a VCSEL with a bonded copper substrate (Cu-Sn bonding) and SiO_2_ current confinement layer (defined as Structure B) was also studied, as shown in Fig. 6b. Each active region was set to be a heat source with a heat power of 8 mW (heat density ~ 4.16 × 10^15^ W m^−3^) during simulation. The temperature rise of Structures A and B inside the cavity is 8.62 and 16.57 K, and the thermal resistance (*R*_th_) were calculated to be 842 and 1428 K W^−1^, respectively. It suggests that structure A has a 41% improvement in heat dissipation. It is clear that, due to the AlN insulator layer and the electroplated copper plate, the thermal energy can be more effectively conducted to the heat sink.

## Conclusions

In summary, we demonstrated continuous-wave green VCSELs with the lowest threshold current density of 51.97 A cm^−2^ and lasing at 524.0 nm. The main factors for achieving a low threshold can be summarized as the strong localization and high IQE (~ 69.94%) of the self-formed InGaN QDs, and the enhancement of interaction between spontaneous emission and lasing mode by a much short cavity (~ 4.0 λ), with a big coupling factor up to 0.094. The thermal characteristic of VCSELs were improved by utilizing the AlN material as current confinement layer and the electroplated supporting copper plate, with a low thermal resistance of 842 K W^−1^.

### Supplementary Information

Below is the link to the electronic supplementary material.

Supplementary file1 (DOCX 1018 kb)
