# Correction to: Green Vertical-Cavity Surface-Emitting Lasers Based on InGaN Quantum Dots and Short Cavity

**DOI:** 10.1007/s40820-023-01270-8

**Published:** 2023-12-20

**Authors:** Tao Yang, Yan-Hui Chen, Ya-Chao Wang, Wei Ou, Lei-Ying Ying, Yang Mei, Ai-Qin Tian, Jian-Ping Liu, Hao-Chung Kuo, Bao-Ping Zhang

**Affiliations:** 1 Laboratory of Micro/Nano-Optoelectronics, School of Electronic Science and Engineering, Xiamen University, Xiamen, 361005 Fujian People’s Republic of China; 2 Suzhou Institute of Nano-Tech and Nano-Bionics, Chinese Academy of Sciences, Suzhou, 215123 Jiangsu People’s Republic of China; 3 Department of Photonics, National Yang Ming Chiao Tung University, Hsinchu, 30010 Taiwan People’s Republic of China; 4 Semiconductor Research Center, Honhai Research Institute, New Taipei, 220236 Taiwan People’s Republic of China

**Correction to: Nano-Micro Lett. (2023) 15:223** 10.1007/s40820-023-01189-0

In this article the author’s name “Hao-Chung Kuo” was incorrectly written as “Hao-Chung Guo”.

And in the last sentence of the first paragraph of Introduction, the text ‘(20-20)’ should have read ‘(20-21)’. The original article has been corrected.

