# Supplementary material for: Green Vertical-Cavity Surface-Emitting Lasers Based on InGaN Quantum Dots and Short Cavity
Source: Nanomicro Lett. 2023 Oct 9;15:223. doi: 10.1007/s40820-023-01189-0 (PMC10562330; doi:10.1007/s40820-023-01189-0)
Supplement: Supplementary file 1 — Supplementary file1 (DOCX 1018 kb) [file 40820_2023_1189_MOESM1_ESM.docx]

Supporting Information for

**Green vertical-cavity surface-emitting lasers based on InGaN quantum dots and short cavity**

Tao Yang^1^, Yan-Hui Chen^1^, Ya-Chao Wang^1^, Wei Ou^1^, Lei-Ying Ying^1^, Yang Mei^1^*, Ai-Qin Tian^2^, Jian-Ping Liu^2^*, Hao-Chung Guo^3, 4^ and Bao-Ping Zhang^1^*

^1^Laboratory of Micro/Nano-Optoelectronics, School of Electronic Science and Engineering, Xiamen University, Xiamen, 361005, Fujian, P. R. China

^2^Suzhou Institute of Nano-tech and Nano-bionics, Chinese Academy of Sciences, Suzhou, 215123, Jiangsu, P. R. China

^3^Department of Photonics, National Yang Ming Chiao Tung University, Hsinchu, 30010,Taiwan, P. R. China

^4^Semiconductor Research Center, Honhai Research Institute, New Taipei, 220236, Taiwan, P. R. China

*Corresponding author. E-mail: meiyang@xmu.edu.cn; jpliu2010@sinano.ac.cn; [bzhang@xmu.edu.cn](mailto:bzhang@xmu.edu.cn)

**S1 Fabrication of VCSEL**

1. **Thickness and topography of the AlN insulating layer and the electroplated copper**

The thickness and topography of AlN have also been our concern in the fabrication of VCSEL. The AlN thickness is about 75 nm. Figure S1(a) shows the 10 × 10 μm^2^ AFM image of AlN, which has a small RMS with 777.6 pm. It indicated that the surface is uniform and flat of the AlN layer.

The thickness and compactness of copper layer are important to the heat dissipation of VCSEL. A proper thickness of copper is needed to provide good support for the epilayers and be a heat sink to conduct the heat. For our VCSEL, the thickness of copper plate is generally 200~300 μm. As shown in Fig. S1(b), the thickness of copper plate in this study is about 205 μm. The cross-section SEM image illustrated in Fig. S1(c), exhibits good compactness without cracks or voids between the copper and Cr/Au layer, which ensures the heat dissipation performance of VCSEL.


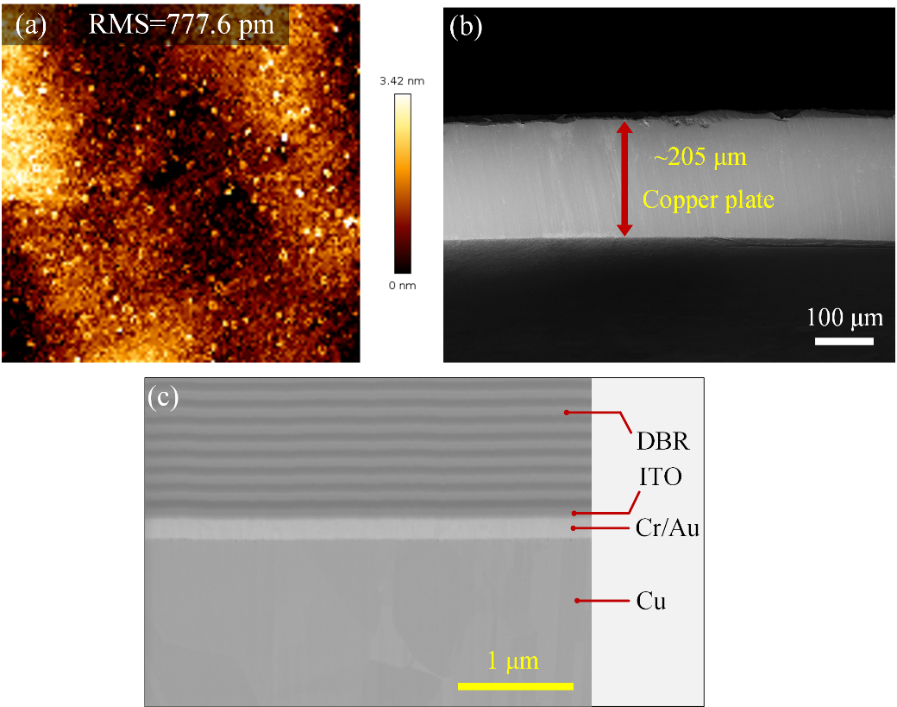


**Fig. S1 (a)** 10 × 10 μm^2^ AFM image of AlN layer. The cross-section SEM image of **(b)** copper plate and **(c)** VCSEL

**S2 Cavity Control of VCSEL**

The short cavity length is realized by using a combination of ICP (fast thinning) and CMP (slow thinning).

(1) ICP thinning. After laser lift-off, the n-face epilayer of the sample was first smoothed by CMP. Then, the epilayer was thinned by ICP etching. Subsequently, the epilayer thickness was thinned and precisely controlled again by CMP.

(2) CMP thinning. The alkaline polishing solution was used to oxidize GaN to Ga_2_O_3_, which is easier to polish. Then, the thickness of epilayers can be precisely controlled by adjusting the sample rotation speed, the impressed pressure on the sample, and the component of the polishing solution (as shown in Fig. S2(a)). It is worth noting that the CMP process needs to be accompanied by a thickness measurement of epilayer (Thin-film interferometry method) until reaches the target thickness. By this method, a smooth surface with RMS less than 300 pm can be obtained as shown in Fig. S2(b).

For stress control, the stress in the epilayer is released and became small after laser lift-off, as demonstrated in our previous work [1]. It thus, has little effect on the subsequent CMP process.


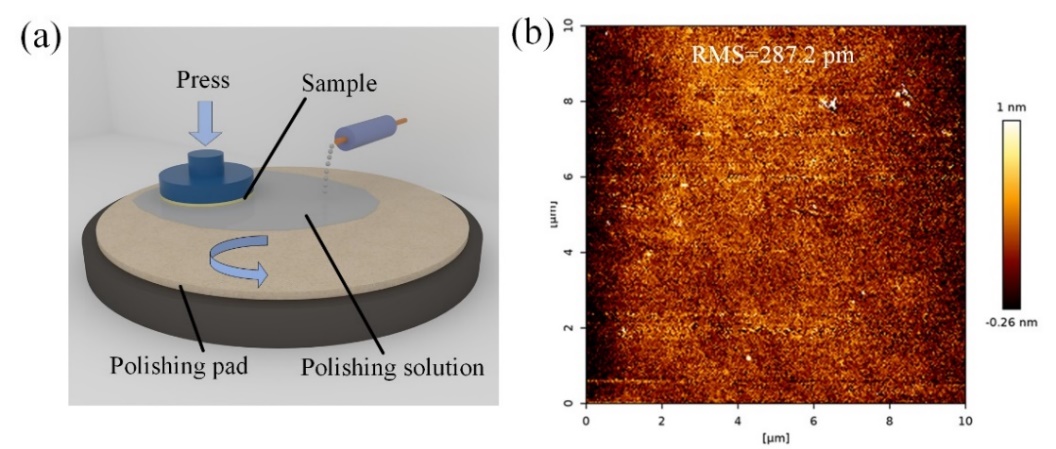


**Fig. S2** **(a)** Schematic of the CMP process **(b)** 10 × 10 μm^2^ AFM image of the epilayer surface after CMP

**S3 Measurement of Cavity Length**

To obtain a precise value of cavity length, the Confocal Laser Scanning Microscope (CLSM) and Stylus Profiler were used to observe and measure the thickness of the cavity, as illustrated in Fig. S3.


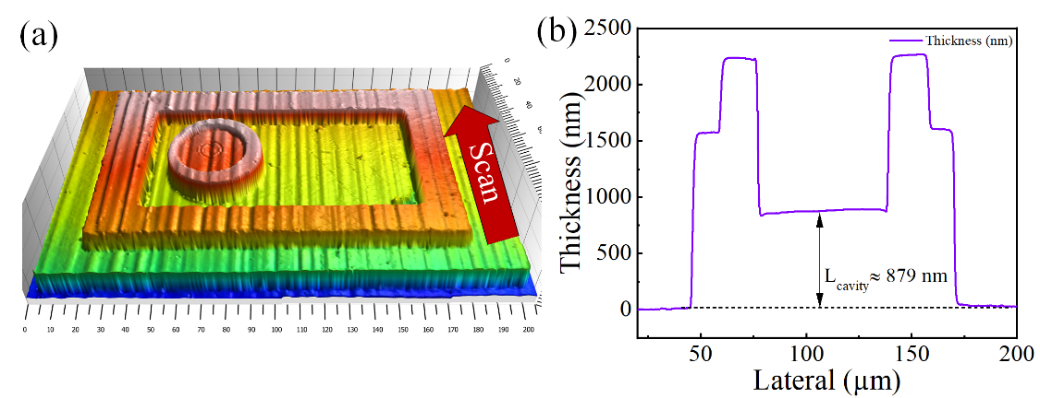


**Fig. S3** **(a)** CLSM image and **(b)** Stylus Profiler measurement result image of VCSEL

**Supplementary References**

[1] S. Yang et al., GaN-based green resonant-cavity light-emitting diodes with Al mirror and copper plate. Opt. let., 47(11), 2858-2861(2022). https://doi.org/10.1364/OL.458088
